# How does the two-child policy affect the sex ratio at birth in China? A cross-sectional study

**DOI:** 10.1186/s12889-020-08799-y

**Published:** 2020-05-27

**Authors:** S. L. Fan, C. N. Xiao, Y. K. Zhang, Y. L. Li, X. L. Wang, L. Wang

**Affiliations:** 1 Hebei Women and Children’s Health Center, Shijiazhuang, 050000 Hebei China; 2 Department of Obstetrics and Gynecology, Hebei General Hospital, No. 348 Heping Road, Shijiazhuang, 050051 Hebei China

**Keywords:** Sex ratio at birth, One-child policy, Two-child policy, Population policy

## Abstract

**Background:**

The One-Child Policy led to the imbalance of the sex ratio at birth (SRB) in China. After that, Two-Child Policy was introduced and gradually liberalized at three stages. If both the husband and wife of one couple were the only child of their parents, they were allowed to have two children in policy (BTCP). If only one of them was the only child, they were allowed to have two children in policy (OTCP). The Universal Two-Child Policy (UTCP) allowed every couple to have two children. The objective of this study was to explore the changing trend of SRB at the stages of Two-Child Policy, to analyze the effect of population policy on SRB in terms of maternal age, delivery mode, parity, maternal education, delivery hospital, and to figure out what factors have greater impact on the SRB.

**Methods:**

The data of the study came from Hebei Province Maternal Near Miss Surveillance System, covered the parturients delivered at 28 gestation weeks or more in 22 hospitals from January 1, 2013 to December 31, 2017. We compared the SRB at different policy stages, analyzed the relationship between the SRB and population policy by logistic regression analysis.

**Results:**

Total 270,878 singleton deliveries were analyzed. The SRB, 1.084 at BTCP, 1.050 at OTCP, 1.047 at UTCP, declined rapidly (χ^2^ = 15.97, *P* < 0.01). With the introduction of Two-Child Policy, the percentage of parturients who were 30–34, ≥35 years old rose significantly, and the percentage of multiparous women increased significantly (40.7, 47.2, 56.6%). The neonatal mortality declined significantly (8.4‰, 6.7‰, 5.9‰, *χ*^*2*^ *=* 44.49, *P* < 0.01), the mortality rate of female infant gradually declined (48.2, 43.7, 43.9%). The logistic regression analysis showed the SRB was correlated to the three population policy stages in terms of maternal age, delivery mode, parity, maternal education, delivery hospital.

**Conclusions:**

The SRB has declined to normal level with the gradually liberalizing of Two-Child Policy in China. Advanced maternal age, cesarean delivery, multiparous women, middle level education, rural hospital are the main factors of effect on the decline of the SRB.

## Background

China has the largest population in the world, total 1,395,380,000 people according to the Sixth Population Census in 2010 [1]. In order to control the population growth rate and reduce the fertility rate, Chinese government implemented the One-Child Policy since 1979, a couple was allowed to have only one child [1]. The One-Child Policy has stopped the births of 400 million babies, and therefore, reduced the birth rate [2]. China’s birth rate has dropped significantly, namely, to a level between 1.5 and 1.7 births per woman, and it has remained the same until now [3]. Meanwhile, the One-Child Policy has brought about some problems, such as highly skewed sex ratio at birth, high sex ratio, ageing of population, pension fund deficiency, shortage of labor force, high Cesarean Delivery (CD) rate and rise of depression of single men that could not find a wife, and etc. [2–6]. The One-Child Policy have profound and lasting influence on China’s population, economy, society, and the health of the Chinese people etc. [2, 7, 8]. Since October 2015, Chinese government implemented the Universal Two-Child Policy, the 36-year One-Child Policy came to an end. The One-Child Policy has led to an increased imbalance in the sex ratio at birth (SRB). The SRB is defined as the ratio of newborn boys to newborn girls [9], and the natural SRB should be kept at 1.05 [10], which means the natural ratio of newborn boys to newborn girls should be 1.05. The SRB peaked at 1.21 in the year of 2005 in china, even rose to 1.40 in some rural areas of central China after the implementation of One-Child Policy [3, 11]. The imbalance of SRB is a global problem, it not only occurs in Asian countries, but also in other countries, such as the United States and Australia [12–18]. Some cultures have traditionally more preference of the birth of boys over the birth of girls [17]. In some Asian and Eastern European countries, such as China, South Korea, India, and Azerbaijan, the sex-selective abortions have resulted in a skewed SRB. The Government of India launched a nationwide program named Beti Bachao Beti Padhao (B3P), strictly prohibiting sex-selective abortion, and as a result, the SRB in Haryana, India decreased from 1.21 in January, 2005 to 1.11 in September, 2016, but still higher than 1.05 [16]. The Chinese government has taken various policies and measures to reduce the SRB, for example, by providing equal work opportunities for men and women, allowing couples in rural areas to have the second child if their first child is a girl, and strictly prohibiting prenatal sex selection, but the SRB remains skewed [2, 3, 19]. But with the implementation of Two-Child Policy, the SRB has declined gradually. The decline of SRB can be attributed to the factors such as policy, economic development, culture, residential area, stress, hormonal variation etc. [3, 20–24]. Is there any relationship between SRB and Two-Child Policy of China?

Hebei Province, a moderately developed area, is located in North China. Hebei Province has a population of 75,195,200, the sixth largest province in population according to China’s population census [1]. The One-Child Policy in Hebei Province was introduced in 1979 and ended in 2016. From April 28, 2011 to May 30, 2014, if both the husband and wife of one couple were the only child of their parents, then the couple were allowed to have Two Children in Policy (BTCP). From May 30, 2014 to January 1, 2016, if only the husband or wife of one couple was the only child of their parents, the couple were also allowed to have Two Children in Policy (OTCP). The Universal Two-Child Policy (UTCP) allowed every couple to have two children since January 1, 2016. The objective of this study was to explore the changing trend of SRB at the three stages of Two-Child Policy that were liberalized gradually, and to analyze the effect of population policy on SRB in terms of the subgroups such as maternal age, delivery mode, parity, maternal education, the location of delivered hospital, and to figure out what factors had greater influence on the SRB after the implementation of Two-Child Policy.

## Methods

### Data collection

We utilized the data collected through Hebei Province Maternal Near Miss Surveillance System (HBMNMSS), which covered all the births in 22 hospitals during the period from January 1, 2013 to December 31, 2017. These 22 hospitals include 7 urban hospitals and 15 rural hospitals in 10 cities of Hebei Province, and the number of annual deliveries in each hospital is more than 1000. Doctors in charge of the patients collected the data and reported to the system through internet. The data included delivery date, delivery mode, maternal education, marital status, maternal age, gestational age at delivery, parity, single or multiple pregnancy, neonatal status, baby sex, birth weight of the baby, hospital location. The data was obtained by means of questionnaires. HBMNMSS was managed by Hebei Women and Children’s Health Center, the quality control of the data was carried out by its staff. Every 6 months, they went to the hospitals to check the accuracy and completeness of the data. Women with singleton pregnancies were included in this study. Women of multiple pregnancies, or of less than 28 gestational weeks, or missing the data of baby sex, gestational age, singleton or multiple delivery and parity were excluded. Ethic approval was given by Hebei Women and Children’s Health Center.

### Definition of variables

According to the course of the population policy, the three stages were defined as BTCP (January 1, 2013 - May 29, 2014), OTCP (May 30, 2014 - December 31, 2015) and UTCP (January 1, 2016 - December 31, 2017). The deliveries ≥28 gestational weeks in HBMNMSS were enrolled, gestational weeks was based on the date of last menstrual period or ultrasonographic findings [25]. The SRB = n (male) / n (female), was defined as the ratio of newborn boys to newborn girls. We stratified the maternal age into different subgroups according to the reference [26] (< 20, 20–24, 25–29, 30–34, ≥35 years old), and also stratified the following factors into different subgroups, such as delivery mode (vaginal delivery, cesarean delivery), maternal education (high level (college and above), middle level (middle school), primary level (primary school and below)), parity (nulliparous (parity< 1), multiparous (parity≥1)), and delivery hospitals (located in urban or rural areas).

### Statistical analysis

The study of birth sex is expressed in two different types of variables, sex ratio at birth (SRB) for continuous variable and rate of male birth for categorical variable. Data description was presented as mean ± standard deviation (Mean ± SD) or median (interquartile ranges (IQR)) for continuous variables and percentages for categorical variables. Group difference was analyzed using One-way analysis of variance (ANOVA) for normally distributed variables and Kruskal-Wallis H test for non-normally distributed variables. Chi-square test was used for categorical variables as group comparison. Multivariable logistic regression model was used to assess the relationship between population policy stages (BTCP, OTCP and UTCP) and male birth with adjustment for maternal age, delivery mode, parity, maternal education and delivery hospital. To explore the relationship between population policy and male birth percentage in different demographic characteristic, subgroup analysis was performed based on subgroup in maternal age, delivery mode, parity, maternal education and delivery hospital. Unadjusted Odds Ratio (OR) and adjusted OR and 95% Confidence Interval (CI) were used to expressed the association in multivariable logistic models with BTCP as control (OR = 1.00). All statistical tests of hypotheses will be two sided and criterion for statistical significance is α = 0.05. Statistical analyses were done with SPSS version 21.0 software (IBM Corp, Armonk, NY).

## Results

From 2013 to 2017, there were 277,925 deliveries at ≥28 gestational weeks in 22 hospitals according to HBMNMSS. Among them, 2912 deliveries that missed the information about baby sex, gestational age, singleton or multiple pregnancy and parity were excluded, and 4091 twin pregnancies, 40 triplet pregnancies, and 1 quadruplet pregnancy were excluded. Total 270,878 singleton deliveries were included and analyzed in this study. The SRB is the ratio of newborn boys to newborn girls at birth. The SRB was 1.084 at BTCP stage, 1.050 at OTCP stage, 1.047 at UTCP stage, it declined rapidly at the three stages (*χ*^*2*^ *=* 15.97, *P* < 0.01).

### The characteristics of demographics and obstetrics

As we showed in Table 1, with the gradually liberalizing of the Two-Child Policy (BTCP, OTCP, UTCP), the age of parturient increased, the percentage of parturient who was at the age of 30–34, and ≥ 35 years old increased significantly, there were more women of advanced maternal age giving birth. The percentage of CD has declined significantly. After Two-Child Policy, more women gave birth to their second child, the multiparous women increased significantly, from 40.7% at BTCP stage, to 47.2% at OTCP stage, and 56.6% at UTCP stage. The women with college and above education increased significantly. The parturients who delivered in urban hospital increased. There was statistically significant difference in the average birth weight, but the regression analysis showed the average birth weight was not related to the SRB at different stages of population policy (Pearson’s correlation coefficient, r = 0.002, *P* = 0.246). The neonatal mortality has declined significantly (8.4‰, 6.7‰, 5.9‰, *χ*^*2*^ *=* 44.49, *P* < 0.01). The SRB of dead neonatal babies increased significantly at different stages, the percentage of the death of the female babies was 48.2% at BTCP stage, and it decreased to 43.7% at OTCP stage, and 43.9% at UTCP stage, this showed that the proportion of death of female babies has gradually declined (*F* = 30.83, *P* < 0.01).

**Table 1:** The Characteristics of Demographics and Obstetrics

	BTCP *n* = 76,466	OTCP *n* = 77,481	UTCP *n* = 116,931	*F/χ*^*2*^	*P value*
Maternal age (year)^a^	27.2 ± 4.6	27.7 ± 4.7^d1^	28.7 ± 4.6 ^d2,d3^	2708.69	< 0.01
< 20 years^b^	1453(1.9)	1294(1.7)	1277(1.1)	5832.92	< 0.01
20–24 years^b^	19,958(26.1)	16,664(21.5)	17,486(15.0)		
25–29 years^b^	36,188(47.3)	37,128(47.9)	55,035(47.1)		
30–34 years^b^	14,059(18.4)	16,056(20.7)	29,702(25.4)		
≥ 35 years^b^	4800(6.3)	6323(8.2)	13,405(11.5)		
Unknown	8(0.0)	16(0.0)	26(0.0)		
Delivery mode^b^				90.81	< 0.01
Vaginal	35,616(46.6)	37,968(49.0)^d1^	57,112(48.8) ^d2,f3^		
Cesarean	40,833(53.4)	39,510(51.0)	59,814(51.2)		
Unknown	17(0.0)	3(0.0)	5(0.0)		
Gestational age (weeks) ^a^	38.9 ± 1.9	38.9 ± 1.7 ^f1^	38.7 ± 1.7 ^d2,d3^	117.36	< 0.01
Parity^b^				4824.41	< 0.01
Nulliparous	45,346(59.3)	40,944(52.8)^d1^	50,793(43.4) ^d2,d3^		
Multiparous	31,120(40.7)	36,537(47.2)	66,138(56.6)		
Education^b^				27.57	< 0.01
College and above	21,564(28.2)	23,096(29.8)^d1^	43,140(36.9) ^d2,d3^		
Middle school	52,462(68.6)	51,683(66.7)	69,754(59.7)		
Primary school and below	1963(2.6)	1767(2.3)	2041(1.7)		
Unknown	477(0.6)	935(1.2)	1996(1.7)		
Marital status^b^				3.97	< 0.05
Married	76,013(99.4)	77,137(99.6)^d1^	116,430(99.6) ^d2,f3^		
Single	438(0.6)	326(0.4)	473(0.4)		
Unknown	15(0.0)	18(0.0)	28(0.0)		
Delivery hospital^b^				401.63	< 0.01
Urban	33,537(43.9)	35,554(45.9)^d1^	56,678(48.5) ^d2,d3^		
Rural	42,928(56.1)	41,924(54.1)	60,249(51.5)		
Unknown	1(0.0)	3(0.0)	4(0.0)		
Baby sex^b^				13.22	< 0.01
Female	36,686(48.0)	37,790(48.8)^d1^	57,133(48.9) ^d2f3^		
Male	39,780(52.0)	39,691(51.2)	59,798(51.1)		
SRB^c, a^	1.084	1.050^d1^	1.047 ^d2,f3^	15.97	< 0.01
Birth weight (kg) ^a^	3.34 ± 0.52	3.33 ± 0.52 ^d1^	3.34 ± 0.51 ^f2,d3^	10.29	< 0.01
Neonatal death^c^	0.912	1.110	1.130		
Male neonatal death^b^	283(44.0)	253(48.5) ^d1^	340(49.7) ^d2,d3^	15.29	< 0.01
Female neonatal death^b^	310(48.2)	228(43.7)	301(43.9)	30.83	< 0.01
Unknown^b^	50(7.8)	41(7.8)	44(6.4)		
Deliveries per day^e^	141(121, 163)	116(90, 162) ^d1^	154(130, 181) ^d2,d3^	47.98	< 0.01

*BTCP:* Both the husband and wife of one couple were the only child of their parents, the couple were allowed to have Two Children in Policy (January 1, 2013 - May 29, 2014); *OTCP:* only the husband or wife of one couple was the only child of their parents, the couple were allowed to have Two Children in Policy (May 30, 2014 - December 31, 2015); *UTCP:* the Universal Two-Child Policy, every couple were allowed to have two children (January 1, 2016 - December 31, 2017). *SRB:* Sex Ratio at Birth. A analysis of variance test of Post Hoc Multiple Comparisons was used for normally continuous variables, and chi-square test for the categorical variables;

^a^The normal distribution continuous data were presented as mean ± standard deviation, the data were compared using independent *t* test (*χ*^*2*^);

^b^The categorical data were presented as numbers (frequencies, %), Chi-square test was used for categorical data (*F*);

^c^n (male) / n (female);

^e^The non-normal distribution continuous data were presented as median (interquartile range), the data were compared using analysis of variance *(F)*;

^d1^Statistically significant when BTCP compared to OTCP (*P* < 0.05);

^d2^Statistically significant when BTCP compared to UTCP (*P* < 0.05);

^d3^Statistically significant when OTCP compared to UTCP (*P* < 0.05);

^f1^Not statistically significant when BTCP compared to OTCP (*P*>0.05);

^f2^Not statistically significant when BTCP compared to UTCP (*P*>0.05);

^f3^Not statistically significant when OTCP compared to UTCP (*P*>0.05)

### Logistic regression analysis

**Fig. 1:**
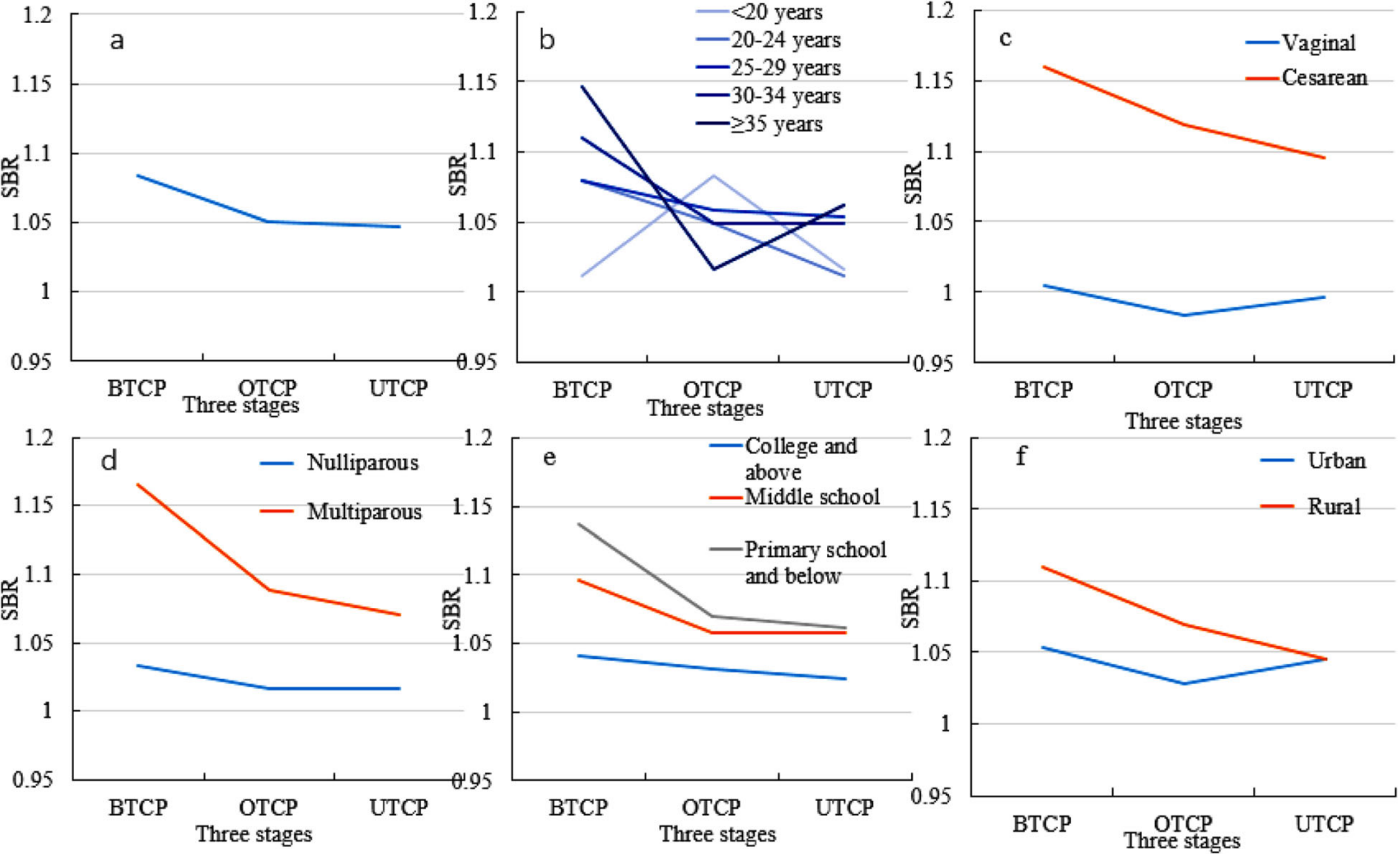
The trend of SRB at three stages of Two-Child Policy (22 hospitals in Hebei Province of China, 2013–2017). **a** The gross SRB at three stages; **b** The SRB in maternal age subgroups at three stages; **c** The SRB in delivery mode subgroups at three stages; **d** The SRB in parity subgroups at three stages; **e** The SRB in maternal education subgroups at three stages; **f** The SRB in urban or rural hospital at three stages. *BTCP:* Both the husband and wife of one couple were the only child of their parents, the couple were allowed to have Two Children in Policy (January 1, 2013 - May 29, 2014); *OTCP:* only the husband or wife of one couple was the only child of their parents, the couple were allowed to have Two Children in Policy (May 30, 2014 - December 31, 2015); *UTCP:* the Universal Two-Child Policy, every couple were allowed to have two children (January 1, 2016 - December 31, 2017). *SRB:* Sex Ratio at Birth

Some factors were statistically different at three stages of population policy, including maternal age, delivery mode, parity, maternal education and delivery hospital. These factors were divided into different subgroups. Figure 1 showed the changing trend of SRB at the three stages of Two-Child Policy in 22 hospitals in Hebei Province of China from 2013 to 2017. Most of factors showed a declined trend in SRB, except for ≥35 years group and urban group. The univariate and multivariate logistic regression analyses were conducted in the subgroups to assess the strength of association between SRB and the population policy.

### Maternal age and SRB

According to our study, there was no statistically significant difference of the SRB at three stages in the subgroups of maternal age < 20, 25–29, ≥35. But the SRB declined significantly in the subgroups of maternal age 20–24(1.079, 1.049, 1.012, *P* < 0.01), 30–34(1.110, 1.049, 1.049, *P* = 0.01). With BTCP stage as a control group, the univariate logistic regression analysis showed that the SRB was significantly correlated to the Two-Child Policy in the subgroups of maternal age 20–24, 30–34, ≥35. After adjusting the factors, multivariate logistic regression analysis showed the SRB was independently correlated to the three stages in the subgroups of maternal age, AOR(95% CI) at OTCP stage was 0.98 (0.94–1.02) at the age of 20–24, 0.94 (0.90–0.99) at the age of 30–34, and 0.89(0.82–0.96) ≥35; AOR(95% CI) at UTCP stage was 0.94(0.90–0.98) at the age of 20–24, 0.94 (0.90–0.98) at the age of 30–34, and 0.93(0.87–0.99) ≥35. The results were showed in Table 2.

**Table 2:** The trend of SRB in the subgroups of maternal age at three stages of Two-Child Policy (22 Hospitals in Hebei Province of China, 2013–2017)

SRB in subgroup	BTCP	OTCP	UTCP	P
< 20 years (SRB)	1.012	1.083	1.016	0.95
20–24 years (SRB)	1.079	1.049	1.012 ^b2^	< 0.01
25–29 years (SRB)	1.079	1.058	1.054 ^b2^	0.06
30–34 years (SRB)	1.110	1.049 ^b1^	1.049 ^b2^	0.01
≥35 years (SRB)	1.146	1.016 ^b1^	1.062 ^b2^	0.10
Unadjusted *OR (95% CI)*				
< 20 years	1.00	1.07(0.91–1.25)	1.01(0.86–1.17)	0.67
20–24 years	1.00	0.97(0.93–1.01)	0.94(0.89–0.98)	< 0.01
25–29 years	1.00	0.98(0.95–1.01)	0.97(0.95–1.00)	0.13
30–34 years	1.00	0.95(0.91–0.99)	0.95(0.91–0.98)	0.02
≥ 35 years	1.00	0.80(0.82–0.95)	0.93(0.87–0.99)	< 0.01
Adjusted *OR (95% CI)*				
< 20 years	1.00	1.08(0.92–1.26)	1.02(0.87–1.19)	0.65
20–24 years	1.00	0.98(0.94–1.02)	0.94(0.90–0.98)	0.01
25–29 years	1.00	0.98(0.95–1.01)	0.97(0.95–0.99)	0.12
30–34 years	1.00	0.94 (0.90–0.99)	0.94(0.90–0.98)	< 0.01
≥ 35 years	1.00	0.89(0.82–0.96)	0.93(0.87–0.99)	< 0.01

*BTCP:* Both the husband and wife of one couple were the only child of their parents, the couple were allowed to have Two Children in Policy (January 1, 2013 - May 29, 2014); *OTCP:* Only the husband or wife of one couple was the only child of their parents, the couple were allowed to have Two Children in Policy (May 30, 2014 - December 31, 2015); *UTCP:* the Universal Two-Child Policy, every couple were allowed to have two children (January 1, 2016 - December 31, 2017). *SRB:* Sex Ratio at Birth (n (male) / n (female)); *OR:* Odds Ratio; *95% CI: 95%* Confidence Intervals

A chi-square test was used in the SRB at the three stages of Two-Child Policy. BTCP acted as a control, Univariate logistic regression was used to examine the strength of association between the SRB and the Two-Child Policy, was showed in Unadjusted *OR* (*95% CI*); After adjusting the factors such as delivery mode, parity, maternal education, delivery hospital, multivariate logistic regression analysis was used, and was showed in Adjusted *OR* (*95% CI*)

^b1^Statistically significant when BTCP compared to OTCP;

^b2^Statistically significant when BTCP compared to UTCP

### Delivery mode and SRB

According to the delivery mode, the parturients were divided into two subgroups: vaginal delivery and cesarean delivery (CD). The SRB of the subgroup of vaginal delivery was lower than that of the subgroup of CD, and there was no statistically significant difference at the three stages. In the subgroup of CD, the SRB declined significantly, from 1.160 to 1.119 and then to 1.096, and much higher than that of the subgroup of vaginal subgroup at the three stages. Univariate logistic regression analysis showed the SRB was significantly correlated to the Two-Child policy in this subgroup. After adjusting the factors such as maternal age, parity, maternal education and delivery hospital, multivariate logistic regression analysis also showed significant correlation between the SRB and the Two-Child Policy in the subgroup of CD. With BTCP stage as a control group, AOR(95% CI) was 0.96(0.93–0.99) at OTCP stage, 0.94(0.92–0.97) at UTCP. The results were showed in Table 3.

**Table 3:** The trend of the SRB in the subgroup of delivery mode at the three stages of Two-Child Policy (22 Hospitals in Hebei Province of China, 2013–2017)

SRB in subgroup	BTCP	OTCP	UTCP	*P*
Vaginal delivery (SRB)	1.004	0.984	0.996	0.65
Cesarean delivery (SRB)	1.160	1.119	1.096	< 0.01
Unadjusted *OR (95% CI)*				
Vaginal delivery	1.00	0.98(0.95–1.01)	0.99(0.97–1.02)	0.37
Cesarean delivery	1.00	0.97(0.94–0.99)	0.95(0.92–0.97)	< 0.01
Adjusted *OR (95% CI)*				
Vaginal delivery	1.00	0.98(0.95–1.01)	0.98(0.95–1.01)	0.24
Cesarean delivery	1.00	0.96(0.93–0.99)	0.94(0.92–0.97)	< 0.01

*BTCP:* Both the husband and wife of one couple were the only child of their parents, the couple were allowed to have Two Children in Policy (January 1, 2013 - May 29, 2014); *OTCP:* only the husband or wife of one couple was the only child of their parents, the couple were allowed to have Two Children in Policy (May 30, 2014 - December 31, 2015); *UTCP:* the Universal Two-Child Policy, every couple were allowed to have two children (January 1, 2016 - December 31, 2017). *SRB:* Sex Ratio at Birth (n (male) / n (female)); *OR:* Odds Ratio; *95% CI: 95%* Confidence Intervals

A chi-square test was used in the SRB at the three stages of the Two-Child Policy. BTCP acted as a control, Univariate logistic regression analysis was used to examine the strength of association between the SRB and the Two-Child Policy, and was showed in Unadjusted *OR* (*95% CI*); After adjusting the factors such as maternal age, parity, maternal education, delivery hospital, multivariate logistic regression analysis was used, and was showed in Adjusted *OR* (*95% CI*)

### Parity and SRB

According to parity, the parturients were divided into nulliparous and multiparous subgroup. The SRB significantly declined in the multiparous subgroup at the three stages, from 1.165 to 1.088 and then to 1.070, but the SRB showed no statistically significant difference in nulliparous subgroup. The SRB of multiparous subgroup was higher than that of the nulliparous subgroup at the three stages. Logistic regression analysis showed that the SRB of nulliparous parturients was not correlated to the Two-Child Policy. But there were significant correlation between the SRB and the Two-Child Policy in multiparous parturients by univariate and multivariate logistic regression analysis. With BTCP stage as a control group, AOR(95% CI) was 0.94 (0.91–0.96) at OTCP stage, 0.92(0.90–0.95) at UTCP stage. The results were showed in Table 4.

**Table 4:** The trend of SRB in the subgroup of parity at the three stages of Two-Child Policy (22 hospitals in Hebei Province of China, 2013–2017)

SRB in subgroup	BTCP	OTCP	UTCP	*P*
Nulliparous (SRB)	1.033	1.016	1.016	0.31
Multiparous (SRB)	1.165	1.088	1.070	< 0.01
Unadjusted *OR (95% CI)*				
Nulliparous	1.00	0.99(0.96–1.01)	0.99(0.96–1.01)	0.51
Multiparous	1.00	0.93(0.91–0.96)	0.92(0.89–0.94)	< 0.01
Adjusted *OR (95% CI)*				
Nulliparous	1.00	0.99(0.96–1.02)	0.99(0.97–1.02)	0.74
Multiparous	1.00	0.94(0.91–0.96)	0.92(0.90–0.95)	< 0.01

*BTCP:* Both the husband and wife of one couple were the only child of their parents, the couple were allowed to have Two Children in Policy (January 1, 2013 - May 29, 2014); *OTCP:* only the husband or wife of one couple was the only child of their parents, the couple were allowed to have Two Children in Policy (May 30, 2014 - December 31, 2015); *UTCP:* the Universal Two-Child Policy, every couple were allowed to have two children (January 1, 2016 - December 31, 2017). *SRB:* Sex Ratio at Birth (n (male) / n (female)); *OR* Odds Ratio; *95% CI: 95%* Confidence Intervals

A chi-square test was used in the SRB at the three stages of Two-Child Policy. BTCP acted as a control, univariate logistic regression analysis was used to examine the strength of association between the SRB and the population policy, showed in Unadjusted *OR* (*95% CI*); After adjusting the factors such as maternal age, delivery mode, maternal education, delivery hospital, multivariate logistic regression analysis was used, and was showed in Adjusted *OR* (*95% CI*)

### Maternal education and SRB

Most women with higher level education were not allowed to give birth to a second child at the stage of One-Child Policy. At the three stages of Two-Child Policy, there was a statistically significant decline in the SRB only in the women with middle level education, from 1.096 to 1.058 and then to 1.058. There was no statistically significant difference in the SRB in the women with primary and high level education. The SRB of women with primary level education remained higher than that of other level education. Univariate and multivariate logistic regression analysis showed the SRB in the subgroup of women with middle level education was significantly correlated to the Two-Child Policy. With BTCP stage as a control group, AOR(95% CI) was 0.96(0.93–0.98) at OTCP stage, 0.95(0.93–0.98) at UTCP stage. The results was showed in Table 5.

**Table 5:** The trend of SRB in the subgroup of material education at the three stages of Two-Child Policy (22 Hospitals in Hebei Province of China, 2013–2017)

SRB in subgroup	BTCP	OTCP	UTCP	*P*
College and above (SRB)	1.041	1.032	1.024	0.30
Middle school (SRB)	1.096	1.058 ^b1^	1.058 ^b2^	< 0.01
Primary school and below (SRB)	1.137	1.070	1.062	0.27
Unadjusted *OR (95% CI)*				
College and above	1.00	0.99(0.95–1.03)	0.98(0.95–1.02)	0.58
Middle school	1.00	0.96(0.94–0.99)	0.96(0.94–0.99)	< 0.01
Primary school and below	1.00	0.94(0.83–1.07)	0.93(0.82–1.06)	0.49
Adjusted *OR (95% CI)*				
College and above	1.00	0.99(0.96–1.03)	0.98(0.95–1.01)	0.46
Middle school	1.00	0.96(0.93–0.98)	0.95(0.93–0.98)	< 0.01
Primary school and below	1.00	0.90(0.79–1.03)	0.90(0.79–1.03)	0.21

*BTCP:* Both the husband and wife of one couple were the only child of their parents, the couple were allowed to have Two Children in Policy (January 1, 2013 - May 29, 2014); *OTCP:* only the husband or wife of one couple was the only child of their parents, the couple were allowed to have Two Children in Policy (May 30, 2014 - December 31, 2015); *UTCP:* the Universal Two-Child Policy, every couple were allowed to have two children (January 1, 2016 - December 31, 2017). *SRB:* Sex Ratio at Birth (n (male) / n (female)); *OR* Odds Ratio; *95% CI: 95%* Confidence Intervals

A chi-square test was used in the SRB at the three Two-Child Policy stages. BTCP acted as a control, univariate logistic regression was used to examine the strength of association between the SRB and the Two-Child Policy, and was showed in Unadjusted *OR* (*95% CI*); After adjusting the factors such as maternal age, delivery mode, parity, delivery hospital, multivariate logistic regression analysis was used, and was showed in Adjusted *OR* (*95% CI*)

^b1^Statistically significant when BTCP compared to OTCP;

^b2^Statistically significant when BTCP compared to UTCP

### Delivery hospital and the SRB

According to the location of hospital, delivery hospitals were divided into two subgroups: urban hospital and rural hospital. The SRB declined significantly in rural hospitals at the three stages of Two-Child Policy, from 1.110 to 1.070 and then to 1.045. There were no significant difference in urban hospitals. The SRB of rural hospital was higher than that of the urban hospital at the stages of BTCP and OTCP. The SRB of urban hospital was not correlated to the Two-Child Policy, but the SRB of rural hospital was significantly correlated to the Two-Child Policy by univariate and multivariate logistic regression analysis. With BTCP as a control group, AOR(95% CI) was 0.96(0.94–0.99) at OTCP stage, 0.93(0.91–0.96) at UTCP stage. The results were showed in Table 6.

**Table 6:** The trend of SRB in urban and rural hospitals at the three stages of Two-Child Policy (22 hospitals in Hebei Province of China, 2013–2017)

SRB in subgroup	BTCP	OTCP	UTCP	*P*
Urban (SRB)	1.053	1.028	1.045	0.78
Rural (SRB)	1.110	1.070	1.045	< 0.01
Unadjusted *OR (95% CI)*				
Urban	1.00	0.97(0.95–1.00)	0.99(0.97–1.02)	0.08
Rural	1.00	0.97(0.94–0.99)	0.94(0.92–0.97)	< 0.01
Adjusted *OR (95% CI)*				
Urban	1.00	0.97(0.94–0.99)	0.983(0.96–1.01)	0.13
Rural	1.00	0.96(0.94–0.99)	0.934(0.91–0.96)	< 0.01

*BTCP: B*oth the husband and wife of one couple were the only child of their parents, the couple were allowed to have Two Children in Policy (January 1, 2013 - May 29, 2014); *OTCP:* Only the husband or wife of one couple was the only child of their parents, the couple were allowed to have Two Children in Policy (May 30, 2014 - December 31, 2015); *UTCP:* the Universal Two-Child Policy, every couple were allowed to have two children (January 1, 2016 - December 31, 2017). *SRB:* Sex Ratio at Birth (n (male) / n (female)); *OR* Odds Ratio; *95% CI: 95%* Confidence Intervals

A chi-square test was used in the SRB at the three Two-Child Policy stages. BTCP acted as a control, univariate logistic regression was used to examine the strength of association between the SRB and the Two-Child Policy, and was showed in Unadjusted *OR* (*95% CI*); After adjusting the factors such as maternal age, delivery mode, parity, education, multivariate logistic regression analysis was used, and was showed in Adjusted *OR* (*95% CI*)

## Discussion

After 36 years of One-Child Policy, China unveiled the Two-Child Policy. The Chinese government aimed to raise the working-age population, curtail the ageing of the population, and normalize the SRB in the future. Whether the low birth rate is the result of One-Child Policy has led to a debate recently. The results of statistical calculations suggested that One-Child Policy had less significant role in driving down the birth rate, but others acknowledged that the One-Child Policy had numerous negative consequences and had significant impact on childbearing decisions [20, 27]. Evidence shows that China has entered an era of low birth rate [28], any restriction policies on birth may no longer be necessary. In fact, after the Two-Child Policy, the birth rate did not increase sharply.

In most human populations worldwide, the SRB is close to 1.05 [9, 10]. There are the problems of high SRB in many countries and regions like India, Uganda, Vietnam, Australia and USA [12, 13, 15, 29–35]. High sex ratio have brought about many social, economic and psychological problems, such as increased mortality in adult male, reduced proportion of rural male marriage, increased depression and suicide in male, and even increased terrorism [4, 36, 37]. The reasons for high sex ratio are complicated, mainly because of sex-selective abortion, especially using ultrasound technology to judge the sex of the fetus before delivery, or the poor care of the baby girl after birth, etc. [18, 38–40]. Boys enjoy more opportunities for work, education, and take more responsibilities for supporting their parents [2, 14, 30, 38, 40]. Other socioeconomic and biological factors such as economic depression, famine, ownership of dwelling, assisted reproductive technologies, and temperature might also affect the SRB [31, 40–48]. Even some festivals can affect the SRB [49, 50]. Socioeconomic factor may be a important factor for SRB at racial, national and global levels. Improving economy lead to increasing education, which in turn tends to lower birth rate in association with a declining SRB. The global correlation of health indicators with SRB suggests that SRB may be a useful and sentinel health and socioeconomic indicator [47, 48, 51]. SRB was a statistically significant variables related to Gross Domestic Product (GDP) per capita, childhood mortality, maternal mortality, life expectancy, overall birth rate, human development index, population, mean years in education [27].

Chinese national census showed that the SRB in China experienced a long imbalance since the implementation of One-Child Policy in 1980s, and it reached 1.202 in 2004. From 2008, China’s SRB fell down gradually. A study of 5,338,853 deliveries in China from 2012 to 2015 showed that the SRB was 1.110 in 2012, 1.102 in 2013, 1.088 in 2014, and 1.095 in 2015, but it was still higher than the normal value [19]. In the past 30 years, Chinese government has taken many policies and measures to control the increased sex ratio, for example, to provide equal work opportunities for men and women, allow rural couples with a girl to have a second child, strictly prohibit prenatal sex identification and sex-selective abortion for more than 14 weeks of gestation without medical indications, and so on [38, 39]. The SRB has dropped significantly, not only because of the prohibition of sex-selective abortion, but also because of economic development, more education and work opportunities for women, the changing of birth concept, and the Two-Child Policy. Our study showed that the Two-Child Policy was the important factor related to the decline of SRB.

This study covers 5 years from 2013 to 2017, from the stage of BTCP, to the stage of OTCP, and then to the stage of UTCP when the Two-Child Policy was introduced and liberalized gradually. The data was collected from 22 hospitals in 10 cities of Hebei Province, located in the central and eastern regions of China. The SRB declined gradually with the liberalizing of the Two-Child Policy, it was 1.084 at BTCP stage, 1.050 at OTCP stage, and 1.047 at UTCP stage. The SRB reached a balance, the number of births has increased significantly after the Two-Child Policy. The average birth weight was about 3.3kgs at three different stages, and the birth weight was not related to the SRB. The neonatal death rate has declined, the proportion of female neonatal death has decreased from 48.2 to 43.9% after Two-Child Policy, which indicated better health care for girls than before.

After the Universal Two-Child Policy, many women gave birth to the second child which was prohibited before, women giving birth at this stage were more likely to be multiparous, especially for those older than 35 years old [52]. From 2013 to 2017, the average maternal age increased, the proportion of parturients ≥30 years old increased from 24.7% at BTCP stage to 36.9% at UTCP stage. With the adjustment of population policy, the SRB has a downward trend, but the biggest decline of SRB is in women ≥30 years old, especially in women ≥35 year old, the SRB was 1.146 at BTCP stage, dropped sharply to 1.062 at UTCP stage. Even after excluding the confounding factors, the women ≥30 year old were still the main factor in the decline of SRB. The SRB was close to normal when women gave birth to the first child, while many women have a stronger willing to have a boy when giving birth to her second child. At the stage of One-Child Policy, in rural areas, if the first child was a girl, the government allowed the couple to have a second child. Some couples kept having babies until the first male baby was born [53]. This rule also resulted in the increased SRB of the second child and increased the gross SRB. After the Universal Two-Child Policy was adopted, this rule was terminated, and the gross SRB swayed back to normal. Women of advanced age increased the rate of complicated pregnancy, such as infertility, fetal malformation, gestational diabetes, placenta previa, postpartum hemorrhage and hypertensive disorders [36]. An expert consensus of healthcare and a practical guideline of the assisted reproductive technology for women with advanced maternal age were published in China, 2019 [54, 55].

The Two-Child Policy also have effect on delivery mode in Liang’s study [8], the rate of CD declined steadily from 2012 to 2016, reaching an overall hospital-based rate of 41.1% in 2016. The classification of CD was modified after the implementation of Two-Child Policy, thus, the rate of CD in nulliparous women decreased [56], but rate in multiparous women increased [52]. The gross rate of CD did not decline much in this study, just from 53.4 to 51.2%, it was difficult to decline rapidly after the Two-Child Policy, with the high rate of initial CD at the stage of One-Child Policy [57]. Morbidly adherent placenta increased in women who had a previous CD. The management and maternal care of high-risk pregnancies were required to ensure the successful implementation of the Two-Child Policy and to improve the maternal and perinatal outcomes in China [58]. There were more baby boys delivered by CD at the stage of BTCP and UTCP, this might suggest that the choice of CD was related to the baby sex. The SRB of vaginal deliveries was below the normal level of 1.05 at all the three stages (1.004, 0.984, 0.996), while the SRB of CD was above the normal level of 1.05 at all the three stages (1.160, 1.119, 1.096). There is a misconception that CD is safer than vaginal delivery. With son preference, after knowing it was a male fetus by prenatal sex determination, couples choose the mode of CD to ensure safe birth of male babies. On the contrary, if it was a female fetus, couples may try to have a vaginal delivery, considering giving birth later. This was one of the manifestation of sex selection. Prenatal sex selection has reduced the mortality of postnatal excessive female babies in South Korea, Armenia, and Azerbaijan, but In India and China, although the absolute number of death of female babies showed a reduction, the mortality of relatively excessive female babies persisted with the increase of prenatal sex selection [14].

After the implementation of One-Child Policy, the SRB was close to normal for the first baby, but it reached 1.46 (1.43 to 1.49) for the second baby, especially in rural areas. The SRB in nine provinces had reached to 1.60 for the second baby, sex selective abortion accounted for almost all the extra male babies [11]. After the implementation of Two-Child Policy, many women in urban areas gave birth to the second child. There were 56.6% multiparous women at the stage of UTCP, much more than 40.7% at the stage of BTCP. The SRB of nulliparous women remained lower than 1.05 at the three stages. Even the SRB of multiparous women declined significantly, it was still much higher than that of the nulliparous women, it was a contributor to the increased SRB. At BTCP stage, the SRB of multiparous women was 1.165, then fell to 1.088 at OTCP stage, and 1.070 at UTCP. This showed that the Universal Two-Child Policy led to the decline of the SRB for the second child. The most male-biased sex ratio and the elevated SRB were found among parity of two or more births, among Indian and Chinese-born mothers, even among the migrants to Australian and the U.S [13, 15].

The education level of the parturients improved. The parturients with college and higher education increased significantly, from 28.2 to 36.9% in 5 years, which indicated more women with high level education had their second babies. The SRB of the women with high level education was the lowest at all stages of Two-Child Policy. More women with high level education were at work, and were the only child of their parents, They had better economic status, but had less willingness to select the sex of the baby. The women with low level education mostly lived in rural areas, the SRB of these women was highest at all three stages. They had no job, no pension insurance, relied on the boy’s pension, so they insisted on giving birth to a boy at least, so the SRB was always at a high level. The parturients with middle level education accounted for 59.7 to 68.6% of the reproductive population, they were the only declined group in SRB, and were the main factor for the decline of SRB. Education played a key role in reducing the degree of gender inequality, the relationship between the SRB and education in India followed an inverted U-shape, the women with middle level education had a higher SRB [59]. The culture of son preference is intricately linked with the economic reality of each couple’s life, education, economic situation, cultural beliefs and affect the entire society, and may lead to the decrease in the country’s SRB [9]. With the urbanization of China, many women with middle level education went to work, and were covered by pension insurance and medical insurance, the SRB of the parturients with middle level education declined.

After the implementation of One-Child Policy, the SRB has been rising for several decades in rural China [9, 11]. Chinese government adopted a number of measures to provide fair working and education opportunities, to preach the ideology of “Giving birth to a boy or girl makes no difference” and change the traditional concepts [2, 3]. With the urbanization of China, many young rural women left home, went to work in cities, and gave birth in urban or rural hospitals, and returned back to work after some time, left their babies with the grandparents in rural areas. The working opportunity brought some income for the rural women, improved their economic situation, and changed their concept of “Preference for boys” and “More birth of sons, more happiness”. At the stage of UTCP, the SRB of rural women has declined to normal level, which is same as that of the urban women. The average lifetime desired birth for the rural women of childbearing age was about 1.71, below the total birth rate at the replacement level [28]. Women’s marriage age, the pecuniary costs of having children, and social security benefits available for rural residents at retirement age, were significantly and negatively related to the willingness of giving birth.

### Strengths and limitations

The five-year study has covered the three stages of Two-Child Policy, which was liberalized gradually. It confirmed that the decline of the SRB was related to the adjustment of population policy, and showed the SRB has returned to normal level after the implementation of Two-Child Policy. The SRB of the women with advanced age, of the multiparous women, of the women with middle education, and of the rural women declined significantly, and had an obvious impact on the decline of the overall SRB. The One-Child Policy was only implemented in China, therefore, this study has a limited reference to other countries. We have not analyzed how the Two-Child Policy made the SRB declined through its impact on birth rate, society, economy, culture and medical care. We have no data before April 28, 2011 when the One-Child Policy was implemented, so the study cannot show the change of SRB completely. We did not consider the period from conception to delivery, and the results might be biased.

## Conclusions

Our study showed that in China, the overall SRB has declined to normal level with the gradually liberalizing of Two-Child Policy after the One-Child Policy. Two-Child policy was a very important factor related to the decline of SRB. Two-Child Policy also resulted in some good results, such as, lower rate of CD, less deaths of baby girl, less sex-selective abortions. There was a lot of work to do for the Chinese government to improve the level of maternal health care, to ensure the welfare of childbearing women, and to reduce the incidence of pregnancy complications.

## Data Availability

The datasets used and/or analyzed during the current study are available from the corresponding author on reasonable request.

## Abbreviations

SRB: Sex ratio at birth
HBMNMSS: Hebei province Maternal Near Miss Surveillance System
BTCP: Both the husband and wife of one couple were the only child of their parents, the couple were allowed to have Two Children in Policy
OTCP: Only the husband or wife of one couple was the only child of their parents, the couple were allowed to have Two Children in Policy
UTCP: The Universal Two-Child Policy, every couple were allowed to have two children
CD: Cesarean Delivery
OR: Odds Ratio
AOR: Adjusted Odds Ratio
95% CI: 95% Confidence Intervals
GDP: Gross Domestic Product

## References

[1] Tabulation on the 2010 Population Census of the People’ Republic of China. http://www.stats.gov.cn/english/Statisticaldata/CensusData/rkpc2010/indexch.htm. Accessed 8 Dec 2019.

[2] Hesketh T, Zhou X, Wang Y (2015). The end of the one-child policy: lasting implications for China. JAMA..

[3] Zeng Y, Hesketh T (2016). The effects of China's universal two-child policy. Lancet..

[4] Zhou XD, Li L, Yan Z, Hesketh T (2013). High sex ratio as a correlate of depression in Chinese men. J Affect Disord.

[5] Liu T (2018). Super-aging and social security for the most elderly in China. Z Gerontol Geriatr.

[6] Mu Y, Li X, Zhu J, Liu Z, Li M, Deng K, Deng C, Li Q, Kang L, Wang Y (2018). Prior caesarean section and likelihood of vaginal birth, 2012-2016, China. Bull World Health Organ.

[7] Barrows SP (2016). China's one-child policy. JAMA..

[8] Liang J, Mu Y, Li X, Tang W, Wang Y, Liu Z, Huang X, Scherpbier RW, Guo S, Li M (2018). Relaxation of the one child policy and trends in caesarean section rates and birth outcomes in China between 2012 and 2016: observational study of nearly seven million health facility births. BMJ..

[9] Lipatov M, Li S, Feldman MW (2008). Economics, cultural transmission, and the dynamics of the sex ratio at birth in China. Proc Natl Acad Sci U S A.

[10] Ein-Mor E, Mankuta D, Hochner-Celnikier D, Hurwitz A, Haimov-Kochman R (2010). Sex ratio is remarkably constant. Fertil Steril.

[11] Zhu WX, Lu L, Hesketh T (2009). China's excess males, sex selective abortion, and one child policy: analysis of data from 2005 national intercensus survey. BMJ..

[12] Egan JF, Campbell WA, Chapman A, Shamshirsaz AA, Gurram P, Benn PA (2011). Distortions of sex ratios at birth in the United States; evidence for prenatal gender selection. Prenat Diagn.

[13] Howell EM, Zhang H, Poston DL (2018). Son preference of immigrants to the United States: data from U.S. birth certificates, 2004-2013. J Immigr Minor Health.

[14] Kashyap R (2019). Is prenatal sex selection associated with lower female child mortality?. Popul Stud.

[15] Edvardsson K, Axmon A, Powell R, Davey MA (2018). Male-biased sex ratios in Australian migrant populations: a population-based study of 1 191 250 births 1999-2015. Int J Epidemiol.

[16] Gupta R, Nimesh R, Singal GL, Bhalla P, Prinja S (2018). Effectiveness of India's National Programme to save the girl child: experience of Beti Bachao Beti Padao (B3P) programme from Haryana state. Health Policy Plan.

[17] Tafuro S, Guilmoto CZ (2019). Skewed sex ratios at birth: a review of global trends. Early Hum Dev.

[18] Chao F, Gerland P (2019). Systematic assessment of the sex ratio at birth for all countries and estimation of national imbalances and regional reference levels. Proc Natl Acad Sci U S A.

[19] Luo Z-C, Huang Y, Tang W, Mu Y, Li X, Liu Z, Wang Y, Li M, Li Q, Dai L (2016). The sex ratio at birth for 5,338,853 deliveries in China from 2012 to 2015: a facility-based study. PLoS One.

[20] Gietel-Basten S, Han X, Cheng Y (2019). Assessing the impact of the “one-child policy” in China: a synthetic control approach. PLoS One.

[21] Song JE, Ahn JA, Lee SK, Roh EH (2018). Factors related to low birth rate among married women in Korea. PLoS One.

[22] James WH, Grech V (2017). A review of the established and suspected causes of variations in human sex ratio at birth. Early Hum Dev.

[23] James WH (2015). Proximate causes of the variation of the human sex ratio at birth. Early Hum Dev.

[24] Bruckner TA, Catalano R, Ahern J (2010). Male fetal loss in the U.S. following the terrorist attacks of September 11, 2001. BMC Public Health.

[25] Obstetrics Subgroup, Chinese Society of Obstetrics and Gynecology, Chinese Medical Association (2014). Diagnosis and therapy guideline of preterm birth (2014). Zhonghua Fu Chan Ke Za Zhi.

[26] Lisonkova S, Potts J, Muraca GM, Razaz N, Sabr Y, Chan WS, Kramer MS (2017). Maternal age and severe maternal morbidity: a population-based retrospective cohort study. PLoS Med.

[27] Goodkind D (2019). Formal comment on “assessing the impact of the ‘one-child policy’ in China: a synthetic control approach”. PLoS One.

[28] Wei J, Xue J (2018). Socioeconomic determinants of rural women's desired fertility: a survey in rural Shaanxi. China..

[29] Manchanda S, Saikia B, Gupta N, Chowdhary S, Puliyel JM (2011). Sex ratio at birth in India, its relation to birth order, sex of previous children and use of indigenous medicine. PLoS One.

[30] Dhatt R, Kickbusch I, Thompson K (2017). Act now: a call to action for gender equality in global health. Lancet..

[31] Wallner B, Fieder M, Seidler H (2012). Ownership of dwelling affects the sex ratio at birth in Uganda. PLoS One.

[32] Subramanian SV, Corsi DJ (2011). Can India achieve a balance of sexes at birth?. Lancet..

[33] Guilmoto CZ, Hoang X, Van TN (2009). Recent increase in sex ratio at birth in Viet Nam. PLoS One.

[34] Jha P, Kumar R, Vasa P, Dhingra N, Thiruchelvam D, Moineddin R (2006). Low female [corrected]-to-male [corrected] sex ratio of children born in India: national survey of 1.1 million households. Lancet..

[35] data S-bsriUcaUn (2016). Son-biased sex ratios in 2010 US census and 2011–2013 US natality data.

[36] Li Q, Deng D (2017). New medical risks affecting obstetrics after implementation of the two-child policy in China. Front Med.

[37] Younas J, Sandler T (2017). Gender imbalance and terrorism in developing countries. J Confl Resolut.

[38] Nie JB (2011). Non-medical sex-selective abortion in China: ethical and public policy issues in the context of 40 million missing females. Br Med Bull.

[39] zhu (2009). China’s excess males, sex selective abortion, and one child policy: analysis of data from 2005 National Intercensus Survey.

[40] West L, Grech V (2019). A systematic search of the factors that influence the sex ratio at birth. Early Hum Dev.

[41] Venero Fernandez SJ, Medina RS, Britton J, Fogarty AW (2011). The association between living through a prolonged economic depression and the male:female birth ratio--a longitudinal study from Cuba, 1960-2008. Am J Epidemiol.

[42] Maalouf WE, Mincheva MN, Campbell BK, Hardy IC (2014). Effects of assisted reproductive technologies on human sex ratio at birth. Fertil Steril.

[43] Bu Z, Chen ZJ, Huang G, Zhang H, Wu Q, Ma Y, Shi J, Xu Y, Zhang S, Zhang C (2014). Live birth sex ratio after in vitro fertilization and embryo transfer in China--an analysis of 121,247 babies from 18 centers. PLoS One.

[44] Song S (2012). Does famine influence sex ratio at birth? Evidence from the 1959-1961 great leap forward famine in China. Proc Biol Sci.

[45] Helle S, Helama S, Jokela J (2008). Temperature-related birth sex ratio bias in historical Sami: warm years bring more sons. Biol Lett.

[46] Grech V (2019). The sex ratio at birth – historical aspects. Early Hum Dev.

[47] Grech V, Calleja N (2019). Multivariate analysis of the correlation of sex ratio at birth with health and socioeconomic indicators. Early Hum Dev.

[48] Grech V (2018). A socio-economic hypothesis for lower birth sex ratios at racial, national and global levels. Early Hum Dev.

[49] Zammit D, Grech V (2019). The effects of thanksgiving, Christmas and Valentine's day on the sex ratio at birth in the United States, 2003-2015. Early Hum Dev.

[50] Grech V, Zammit D (2019). Influence of the super bowl on the United States birth sex ratio. Early Hum Dev.

[51] Grech V (2018). Correlation of sex ratio at birth with health and socioeconomic indicators. Early Hum Dev.

[52] Li HT, Xue M, Hellerstein S, Cai Y, Gao Y, Zhang Y, Qiao J (2019). Association of China’s universal two child policy with changes in births and birth related health factors: national, descriptive comparative study. BMJ..

[53] Grech V, James WH, Lauri J (2018). On stopping rules and the sex ratio at birth. Early Hum Dev.

[54] Pregnancy-induced Hypertension Disease Subgroup, Chinese Society of Obstetrics and Gynecology, Chinese Medical Association (2019). Expert consensus on the pre rectanglepregnancy, pregnancy and intrapartum management of women with advanced maternal age, 2019. Zhonghua Fu Chan Ke Za Zhi.

[55] Jiang L, Chen Y, Wang Q, Wang X, Luo X, Chen J, Han H, Sun Y, Shen H (2019). A Chinese practice guideline of the assisted reproductive technology strategies for women with advanced age. J Evid Based Med.

[56] Zhao J, Shan N, Yang X, Li Q, Xia Y, Zhang H, Qi H (2017). Effect of second child intent on delivery mode after Chinese two child policy implementation: a cross sectional and prospective observational study of nulliparous women in Chongqing. BMJ Open.

[57] Zhang Z, Gu C, Zhu X, Ding Y, Simone S, Wang X, Tao H (2018). Factors associated with Chinese nulliparous women's choices of mode of delivery: a longitudinal study. Midwifery..

[58] Cheng PJ, Duan T (2016). China’s new two-child policy: maternity care in the new multiparous era. BJOG..

[59] Echavarri RA, Ezcurra R (2010). Education and gender bias in the sex ratio at birth: evidence from India. Demography..

